# High-definition infrared thermography of ice nucleation and propagation in wheat under natural frost conditions and controlled freezing

**DOI:** 10.1007/s00425-017-2823-4

**Published:** 2017-12-09

**Authors:** David P. Livingston, Tan D. Tuong, J. Paul Murphy, Lawrence V. Gusta, Ian Willick, Micheal E. Wisniewski

**Affiliations:** 1 USDA-ARS and North Carolina State University, Raleigh, NC USA; 2 North Carolina State University, Raleigh, NC USA; 3 University of Saskatchewan, Saskatoon, Canada; 4 USDA-ARS, Kernersville, WV USA

**Keywords:** *Triticum aestivum*, Crown, Xylem, Radiative cooling, Supercooled

## Abstract

**An extremely high resolution infrared camera demonstrated various freezing events in wheat under natural conditions. Many of those events shed light on years of misunderstanding regarding freezing in small grains.**

Infrared thermography has enhanced our knowledge of ice nucleation and propagation in plants through visualization of the freezing process. The majority of infrared analyses have been conducted under controlled conditions and often on individual organs instead of whole plants. In the present study, high-definition (1280 × 720 pixel resolution) infrared thermography was used under natural conditions to visualize the freezing process of wheat plants during freezing events in 2016 and 2017. Plants within plots were found to freeze one at a time throughout the night and in an apparently random manner. Leaves on each plant also froze one at a time in an age-dependent pattern with oldest leaves freezing first. Contrary to a common assumption that freezing begins in the upper parts of leaves; freezing began at the base of the plant and spread upwards. The high resolution camera used was able to verify that a two stage sequence of freezing began within vascular bundles. Neither of the two stages was lethal to leaves, but a third stage was demonstrated at colder temperatures that was lethal and was likely a result of dehydration stress; this stage of freezing was not detectable by infrared. These results underscore the complexity of the freezing process in small grains and indicate that comprehensive observational studies are essential to identifying and selecting freezing tolerance traits in grain crops.

**Electronic supplementary material:**

The online version of this article (10.1007/s00425-017-2823-4) contains supplementary material, which is available to authorized users.

## Introduction

The response of plants to freezing temperatures has been studied for over a century (reviewed by Wisniewski et al. 2003) and has revealed that freezing in plants is a complex process. Environmental variables, such as soil moisture and temperature fluctuations, as well as physiological and genetic factors within plants, including the presence or absence of intrinsic and extrinsic nucleating agents and structural barriers that inhibit the propagation of ice, are all important factors determining whether a plant survives freezing conditions. These variables all interact continuously in a seemingly stochastic manner making the improvement of freezing tolerance in plants a considerably challenging endeavor.

Histological observations and evaluation of internal tissues, either during freezing or shortly after thawing have been conducted to determine the freezing tolerance of various tissues (Aloni and Griffith 1991; Livingston et al. 2005; 2006, 2013; Olien and Marchetti 1976; Pearce et al. 1998; Stier et al. 2003; Tanino and McKersie 1985; Zamecnik et al. 1994). However, the size of tissues that need to be examined, and the fact that they are embedded in and surrounded by other plant structures, have made the interior of the plant difficult if not impossible to monitor during freezing. A 3D reconstruction technique using histological freeze-fixation was used to infer the location of ice within crowns and suggested the shapes of ice formations differed depending in which tissues the ice was located (Livingston and Tuong 2014). The freeze-fixation technique is destructive however, and thus a real-time analysis cannot be conducted on freezing responses using the technique. Magnetic resonance micro imaging (MRMI) has been used to study freezing in a non-destructive manner in flowers of dogwood (Ishikawa et al. 2016). A detailed analysis of dehydration and freezing in that study indicated that anthers and ovules remained supercooled while other floral tissues either froze or were dehydrated. While a high level of resolution of free and bound or frozen water in internal tissues can be achieved using MRMI analyses, several aspects of this technology, including logistical considerations, access to equipment, and expense make it impractical in many circumstances.

When liquid water freezes it gives up energy in the form of heat. This attribute has been used to monitor the freezing of plant tissues using thermocouples, thermistors, and more recently, infrared thermography (Wisniewski et al. 1997). In that study, an analog-based long-wave, commercial infrared camera was used to observe the initial site of ice formation and subsequent ice propagation in bean leaves, peach and apple flowers, and rhododendron stems. The ability of the ice nucleating-active (INA) bacterium, *Pseudomonas syringae*, to induce freezing in herbaceous plants that would otherwise supercool, was visually confirmed. The role of INA bacteria in inducing freezing in herbaceous was initially documented in earlier studies (see review by Upper and Vali 1995) and formed the basis of controlling INA bacteria populations on plants as a frost-protection management strategy (reviewed by Lindow 1995).

The use of infrared thermography revolutionized the study of freezing in plants (Wisniewski et al. 1997, 2014) and allowed a form of tissue-specific calorimetry that was not previously possible (Livingston 2018). This approach has been used by numerous researchers to study the freezing process in plants and document the role of intrinsic and extrinsic nucleators that induce ice formation and the role of structural barriers in regulating ice propagation (Wisniewski et al. 2008; Ball et al. 2002; Fuller and Wisniewski 1998; Kuprian et al. 2016, Neuner et al. 2010). Hacker and Neuner (2007) further developed the analysis of the IR video data using commercial software to subtract thermal images from the thermal output of subsequent images, labeling their approach “infrared differential thermal analysis” (IDTA). This approach was modeled after the use of differential thermal analysis (DTA) to monitor supercooling in plants (Quamme et al. 1972).

The utilization of infrared thermography to observe the freezing process in plants has progressed from analog systems (Wisniewski et al. 1997), where data were recorded on a videotape, to digital systems (Hacker and Neuner 2007) where the data is recorded in digital files on a computer (Wisniewski et al. 2015). Most recently, high-definition infrared cameras with increased pixel resolution have become available that have significantly increased image clarity. This has resulted in a greater ability to discern details about the freezing process, especially under field conditions.

Laboratory experiments by their nature tend to compromise the environmental complexity that occurs under natural conditions and may affect the freezing process. The question has often been raised as to whether controlled freezing experiments have any relevance to what occurs under natural conditions. Therefore, the purpose of the present study was to use high-definition infrared thermography of wheat plants to compare freezing patterns in the field with experiments conducted under controlled conditions. The analysis spanned a 2-year period and included observations made during fall when plants were in a vegetative state and then during spring, when the same plants had entered a reproductive phase of growth. In both years during spring, a significant freeze event occurred while wheat plants were in their mid-boot growth stage. Importantly, while still video images have been used to illustrate the observations made in this study, readers are strongly encouraged to view the supplementary video files to fully comprehend observations that support the results.

## Materials and methods

### Natural conditions

Four wheat genotypes (Spring-freeze tolerant: Progeny 185, AG South 2056; Not freeze tolerant: Merl, AGSouth 2038) were selected from a larger population of wheat that was previously characterized for vegetative as well as spring-freeze tolerance (Livingston et al. 2016). Seeds were sown at a depth of 3 cm at the Mid-Pines Research station in Raleigh, NC on October 21, 2015 in the first year and in the second year on October 3, 2016. They were replicated twice in 3 m rows in a 3 × 3 m plot with row spacing of 10 cm.

#### Season 1

On January 18, and February 13, 2016, plants in the vegetative state were subjected to an overnight, freeze to − 8.1 and − 8.0 °C, respectively; the events were recorded from 7 p.m. until 7 a.m. the following morning. The same plot experienced a freeze to − 2.4 °C on April 10, 2016 when plants were at mid-boot stage of growth (Feekes 8–9, Zadoks 38–43).

#### Season 2

The winter of 2016–2017 was one of the warmest on record and consequently plants in the vegetative stage did not encounter a significant freeze. Due to the mild winter, the wheat crop in NC, VA, WV, KY, and TN matured 2 weeks to a month earlier than normal. On March 14, 15 and 16, 2017 a three-night, sequential freeze was experienced with the second night encountering a low of − 8 to − 10 °C by 7:30 a.m. at the Mid-Pines station. Many areas of NC, TN and KY experienced colder temperatures on all three nights.

Thermocouples were placed 1–2 mm under the soil surface and inserted into the center of a head within the boot of two separate plants. Thermocouple temperatures were within 1 °C of the temperatures reported by Research IR software. However, thermocouples measured temperatures at the point of insertion, whereas Research IR software measured the average temperature over a specified area. For that reason temperatures in videos measured with thermocouples may not agree with those shown on the graph in Fig. 2.

### IR recording and image processing

In 2015 two medium-resolution (640 × 480 pixels) FLIR T620 infrared cameras were used. One camera was used to record freezing of the entire plot [supplemental video (SV) 1] and the second camera recorded close-up images of individual plants (SV2). In March 2017 a FLIR SC8303, at considerably higher resolution (1280 × 720 pixels), was included with the two T620 cameras (SV3).

In both years continuous video recordings were captured on screen at 30 frames/s using BandiCam software. IR monitoring of freezing began at 8:00 p.m. and ended around 7:00 a.m. the next morning. Air temperatures ranged from 1 or 2 degrees above 0 to − 10 °C. Soil temperatures ranged from 5 to − 5 °C. The recording was also saved using Research IR, FLIR’s in-house recording format. After recording, screen-captured MP4 files were converted to .mov format and edited using Adobe After Effects.

### Controlled conditions

IR monitoring under natural conditions (SV1, 2, 3) was compared to wheat grown and frozen under controlled conditions as described by Livingston et al. (2016). Briefly, wheat seeds (CV Shirley) were planted in cone-tainers and then grown in growth chambers for 5 weeks at 13 °C and then 3 weeks at 3 °C to simulate cold acclimation (CA).

**Fig. 1:**
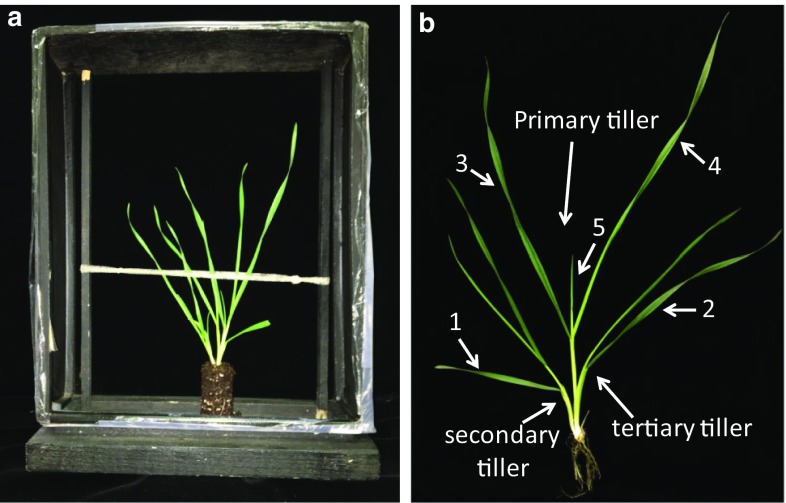
**a** A wooden open-frame structure used to contain wheat plants for freezing. The soil plug from the plant was trimmed to 5 cm so the intact plant would fit inside the frame. Midway up the plant a small loop of nylon hose was used to loosely support the plant during freezing. **b** A cold-acclimated wheat plant with leaves spaced apart to show the different leaves discussed in the study. This plant was 8 weeks old and had been cold-acclimated at 3 °C for 3 weeks prior to testing. It had three tillers; the primary tiller is shown in the center between the secondary and tertiary tillers. Leaf 1 is the oldest leaf on the plant and its sheath surrounds the entire plant; it is the first leaf to emerge from the coleoptile after germination. Leaf 2 is opposite leaf 1 and is the second oldest leaf; its sheath is inside leaf 1; the sheath of leaf 2 also surrounds the entire plant. Leaf 3 is the oldest leaf on the primary tiller and its sheath surrounds the entire primary tiller. The sheath of leaf 4 is inside leaf 3 and is the next youngest leaf. Leaf 5 is the last leaf on the primary tiller that is visible in this image. Leaves 6 and 7 and sometimes leaf 8 (depending on growing conditions) are inside leaf 5 and are only visible upon dissection of the tiller. The apical meristem would be the equivalent of the youngest leaf on the tiller. Different growing conditions would undoubtedly affect the number of leaves within this tiller

To record freezing patterns under controlled conditions, CA plants were removed from cone-tainers. The soil plug containing roots was trimmed to 10 cm and was placed in a 30 (*h*) × 24 (*w*) × 7 (*d*) cm wooden frame, lined with velvet, with a thin nylon band to hold plants upright (Fig. 1a) and placed in a freezer at 0 °C. When the temperature of the soil had equilibrated with the freezer, as evidenced by the soil color becoming similar to the background, the freezer was ramped down to the target temperature at 2 °C/h. It was held at the target temperature for 2 h and then thawed at 4 °C/h.

### Freeze-fixation

The mid and upper portion of the oldest leaf on the primary tiller was removed with scissors inside the freezer just after determining that the leaf had undergone an initial rapid freeze localized in vascular bundles (stage 1 freezing) or a slower, more extensive freeze encompassing the entire leaf (stage 2 freezing). Leaf segments were placed in methanol-based FAA fixative that was at the same temperature as the chamber. The tissue was then processed as described (Livingston and Tuong 2014).

### Dry weights

Dry weights of separate leaves (Fig. 1b) from cold-acclimated plants were measured by weighing before and after drying in an oven at 60 °C for 12 h, to constant weight (Table 1). Leaves were removed by hand (not with razor or scissor) from crowns at a natural separation point near the base of the sheath. In this context “leaves” included the sheath and collar as well as all portions of the leaf proper. Results were analyzed as a one-way analysis of variance using SAS (version 9.3) proc GLM and subjected to a Tukey’s mean separation test.

**Table 1:** Percent water of wheat leaves from oldest to youngest leaf on primary tiller

Leaf	Leaf age	% water
Oldest whole plant leaf	1 (oldest)	85.0a*
Second oldest whole plant leaf	2	82.5ab
Oldest primary tiller leaf	3	80.2bc
Next inside leaf on primary tiller	4	77.6cd
Third inside leaf	5	75.6de
Fourth inside leaf	6	74.3e
Fifth and innermost leaf, above apical meristem	7 (youngest)	67.6f
LSD (0.05)		2.64%

*N* = 6 plants

*Letters adjacent to values within the column that are the same are not significantly different from each other at *P* = 0.05

### Histology

Leaves from CA plants were fixed in methanol-based FAA, dehydrated, paraffin embedded and sectioned at 15 microns as described (Livingston and Tuong 2014). Sections were triple-stained with Saffranin, Fast Green and Orange G, mounted on slides with Permount and photographed with a Canon Rebel T5i camera attached to a Nikon Eclipse 50i. Images were processed in Adobe After Effects as described previously (Livingston et al. 2010).

## Results and discussion

### Fluctuation in air temperature within and directly above the plant canopy

**Fig. 2:**
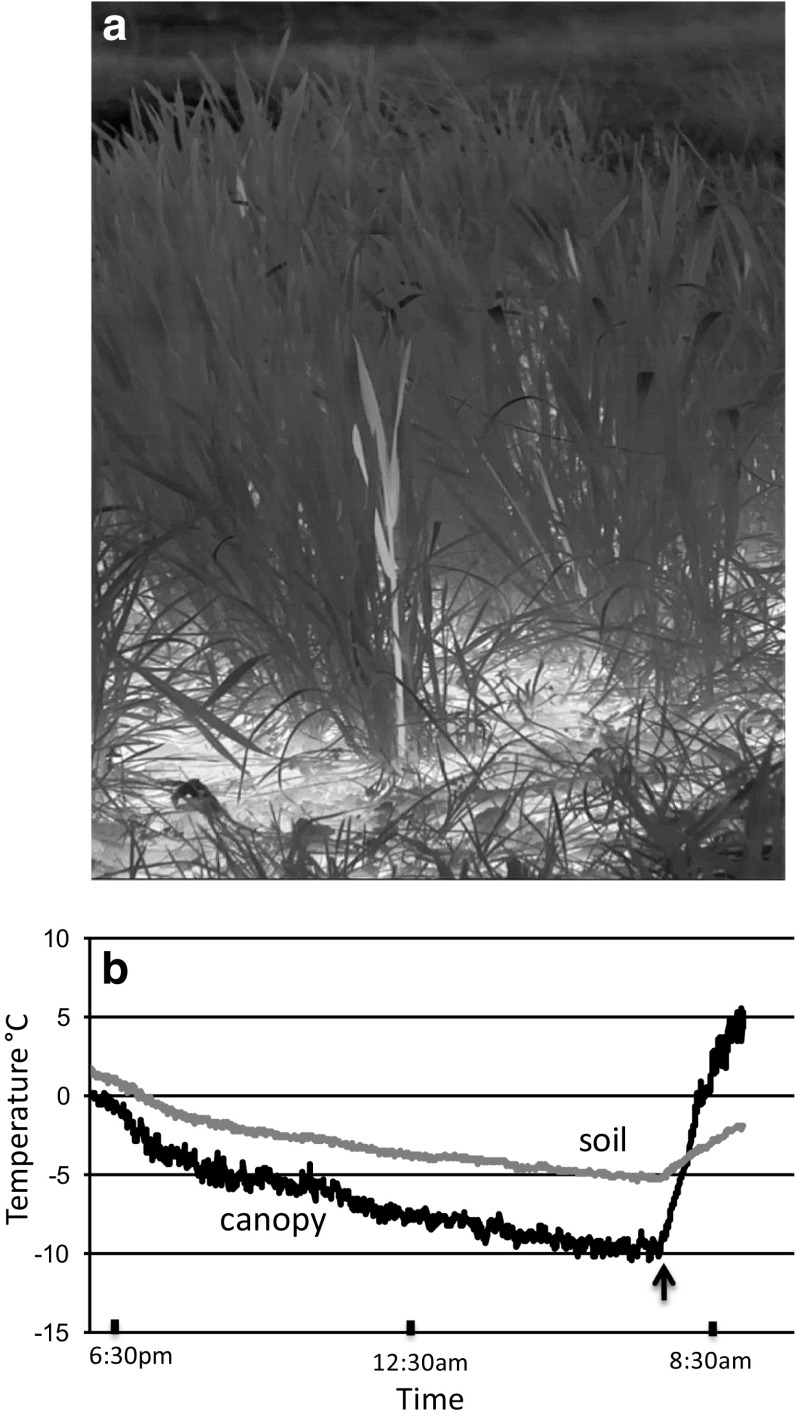
**a** Single frame of wheat in the reproductive phase of growth from supplemental video 3 recorded under natural conditions with a high-definition infrared camera during the night of March 15, 2017. Lighter colors indicate warmer temperatures, Note the lighter colored plant near the center of the image that has frozen. Also note that the color of the soil is a significantly lighter color than the plant canopy indicating a higher temperature than the plants. **b** Soil and canopy temperatures during the night were determined by camera software. Note the greater temperature fluctuation in the canopy in contrast to that in the soil. Also note that the soil was always 1 or 2 degrees warmer than the canopy; by 7:30 a.m. the difference was around 5 °C. The arrow just prior to 8:30 a.m. is the time at which the sun impacted the plot and rapidly increased the temperature of both the canopy and soil (not shown in SV3)

Despite little or no perceptible air movement, the air temperature above and within the plant canopy exhibited a considerable amount of turbulence throughout the night (SV1, SV2, SV3). In 2017, there was a two to three degree fluctuation in canopy temperatures as compared to half a degree at the soil surface (Fig. 2). In some cases, plant movement, indicating micro-gusts of warm or cool air, provided a source for the observed fluctuations but, when no air movement was apparent, the cause of the turbulence was clearly from another source. Periodic waves of warm air can be seen moving up from the soil surface (SV1, SV3) and since soil temperatures were always several degrees above air and canopy temperature (Fig. 2b) it is likely that heat released from the soil converged with air temperature, generating most of the temperature fluctuations observed within the canopy. When the soil surface reached 0 °C, latent heat flux (Arya 2001) from areas of the soil surface that was beginning to freeze was the likely source of temperature turbulence in the canopy. Temperatures at the soil surface were not uniform and there were regions of the soil that froze at different times throughout the night (SV1, SV2, SV3). It is not known if these temperature fluctuations had a direct effect on the freezing events described below.

The continual fluctuations in temperature observed during these natural freezing events suggest that more consideration should be given to how the concept of radiative cooling influences our perception of freezing in plants. With an arguably unlimited supply of heat from the soil and its continual convergence and divergence with air temperature, creating seemingly non-stop turbulence, does strict radiative cooling ever really occur within the plant canopy? Single and Marcellos (1981) emphasize the importance of the “steep temperature gradient in the canopy during a radiation frost” when trying to understand freezing. Our results, under all scenarios, suggest that when the soil is at or near freezing, latent heat flux converging and diverging with cooler air in the canopy will cause significant random fluctuations in temperature gradients (SV3). Therefore, the observed temperature turbulence above the soil and within the plant canopy must be taken into account when considering how radiative cooling (windless night with clear skies) affects the freezing process in plants.

### Overall freezing response

**Fig. 3:**
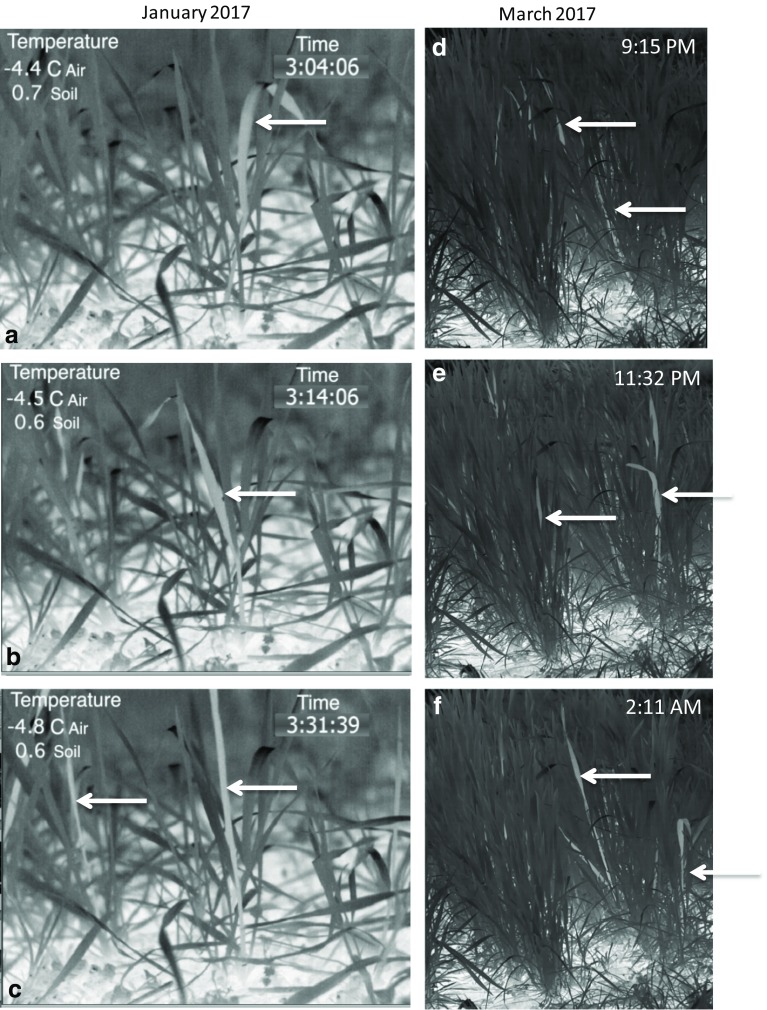
**a**–**c** Three frames of a recording from SV2 of wheat in the vegetative stage of growth during the night of January 18, 2016 with a medium-resolution infrared camera. White arrows indicate individual plants that froze as indicated by their lighter color than the background color. The three frames show leaves freezing independently which occurred throughout the night and is much more obvious in both the wide angle video (SV1) as well as the close view (SV2). The time signature in the upper right corner is the time from the beginning of the recording in hours:minutes:seconds. **d**–**f** Freeze-frames from a high-definition recording (SV3) showing wheat leaves and plants under natural conditions freezing independently from each other (white arrows) identically to plants in a vegetative growth phase (SV1, SV2). This freeze event was recorded during the night of 15 March, 2017 and is from the same video used in Fig. 2a. The reader is encouraged to view supplemental video 3 to better visualize this demonstration of freezing under natural conditions of wheat in a reproductive phase of growth

The wheat plants within the plot monitored with the infrared camera did not freeze uniformly as a unit at a single temperature either in the fall (Fig. 3a–c, and SV1, SV2) or spring (Fig. 3d–f, and SV3). Numerous freezing events were observed throughout the night, over a wide range of temperatures, in both vegetative plants observed in the fall as well as in plants in the reproductive stage in spring (Fig. 3 and SV1, SV2, SV3). The same was true for individual plants, as different leaves froze at different temperatures. The first observable freeze event in March 2017 was at 8:40 p.m. when the air temperature was − 2.3 °C and the soil temperature, as measured by thermocouple, was 2.5 °C (SV3). Each freeze event was localized to a single leaf of an individual plant (SV1, SV2, SV3). Latent heat released from the soil, which contributed to fluctuations in plant canopy temperature, may have partially contributed to the apparently random order of plant freezing. Fuller et al. (2007), using infrared thermography, observed that wheat plants in the reproductive stage froze over a wide temperature range as independent units. They also found that some wheat culms supercooled to temperatures as low as − 15 °C, likely indicating the absence of efficient intrinsic nucleating agents. This underscores the suggestion (Wisniewski et al. 2014) that supercooling is an adaptive mechanism that would allow some plants to escape injury by means of freeze avoidance.

### Age-dependent leaf freezing

**Fig. 4:**
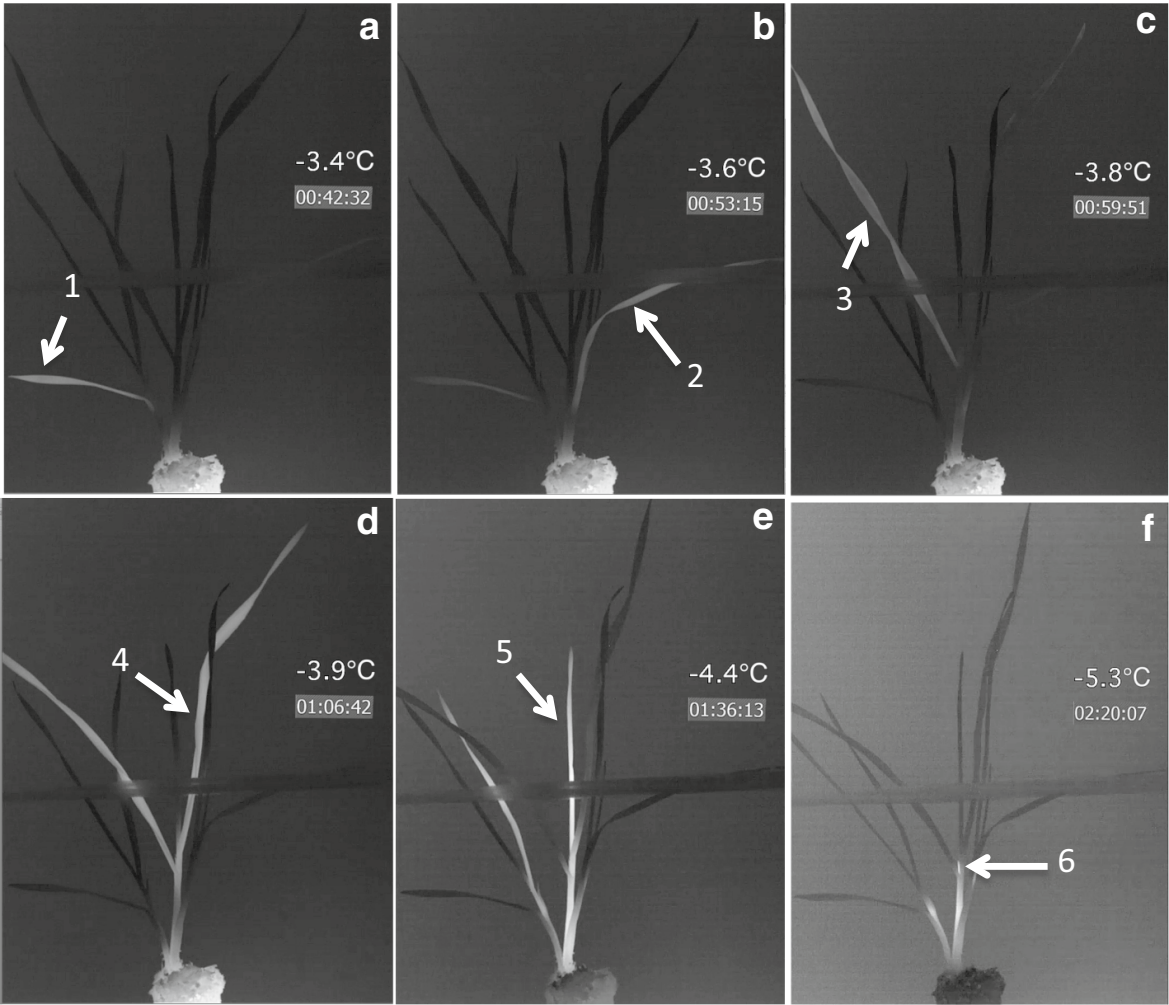
A wheat plant frozen under controlled conditions. Individual frames **a** through **f** are from a high-definition recording (SV4) of wheat in a vegetative stage of growth and demonstrates an age-dependant sequence of leaves freezing. Leaf numbers correspond to the age-sequence in Fig. 1b. Time is lapsed time from the start of the recording in hours:minutes:seconds. Temperatures were recorded by a thermocouple in close proximity to the plant. Note in **f** that only the very tip of leaf 6 is visible. Some of the latent heat from this leaf radiated outward, causing the bottom of the entire primary tiller to become warmer than the surrounding tissue. Also note that leaf 7 is not visible and as indicated by a lack of an exotherm never froze in this freeze test. However, note the exotherm at the base of the tiller to the left signifying freezing of a leaf inside. The reader is encouraged to view supplemental video 4 to better visualize this demonstration of age-dependant freezing in a wheat plant

Analysis of the infrared videos during both natural (SV1, SV2, SV3) and controlled freezing (SV4) indicated that the oldest leaf generally froze first and that freezing progressed to younger leaves as temperatures declined. This was the case in plants that were in a vegetative growth stage (SV2) as well as a reproductive stage (SV3). The age-dependent sequence of freezing was especially obvious under controlled conditions when individual plants could be observed in greater detail (Fig. 4, SV4). Grass leaves are arranged in an alternate, semi-circular pattern on either side of the culm. Each leaf is 180° opposite the next leaf with older leaves in the externally outermost position. The removal of outer leaves reveals that the innermost, youngest leaf is located directly above the apical meristem (Livingston et al. 2013). When the innermost leaves froze, latent heat radiated to the outer leaves and was observed as a diffuse freeze event just above the crown (Fig. 4f and SV4 toward the end of the video). The intensity (amount of heat released), as well as duration of the freezing event, was dependent upon the degree of supercooling that had occurred prior to the onset of freezing. Innermost leaves that froze at colder temperatures (greater degree of supercooling) produced a very bright IR image due to the differential between the temperature of the latent heat released and the surrounding air temperature (SV4).

Hacker and Neuner (2007) reported an “age-dependant freezing pattern” with older leaves freezing before younger leaves in shoots of *Buxus sempervirens*, an evergreen shrub. They also observed a similar sequence of freezing in needles of *Taxus baccata* and in stems of *Fagus sylvatica,* where ice propagation was 56% slower in younger stems than it was in older stems (Hacker and Neuner 2007). Pearce and Fuller (2001) also found that, once roots had frozen, leaves of barley froze in an age-dependant pattern identical to the observations made on wheat plants in the current study.

A potential explanation for the age-dependent sequence of freezing from older (outer) to younger (inner) leaves is the insulating effect that the outer leaves could have on the innermost leaves. This would be similar to the insulating effect described in cauliflower (Tapsell et al. 1990). There is no direct evidence, however, that the inner leaves of wheat plants are any warmer than the outer leaves. It is possible that some of the latent heat released from the freezing of outermost leaves (Fig. 4a, b), which have a higher water content than the inner leaves (Table 1), radiated inward and delayed the freezing of the inner leaves.

**Fig. 5:**
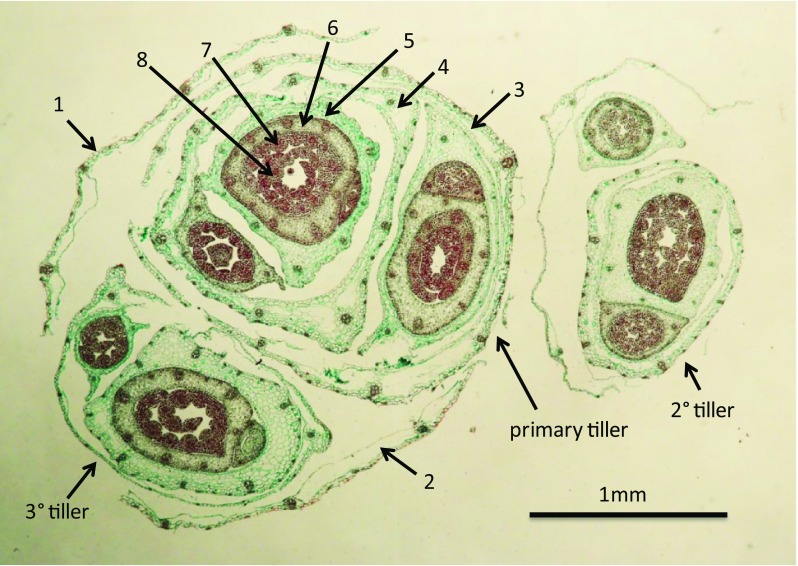
A cross section of an unfrozen, cold-acclimated wheat plant just above the apical meristem, showing the difference in cell sizes between the outer (older) and inner (younger) leaves. Numbers with arrows point to leaves on the primary tiller that correspond to the leaves shown in Fig. 1b. Note the large thin walled cells in the outer leaves (leaves 1–5). These leaves had a significantly higher percentage of water (Table 1) than younger leaves in the center of the tiller; cells in leaves 6–8 were more dense and compact with visibly smaller xylem vessels

The younger, developing leaves with smaller cells (Fig. 5), and lower water content (Table 1), would likely have the propensity to supercool. Additionally, younger leaves had smaller intercellular spaces and less developed xylem which could have hindered the ability of ice to propagate from the base of the plant where ice formation was initiated (see below). Xylem conducting elements have been documented as the main conduit of ice propagation in most plant species (Hacker and Neuner 2007; Pearce 2001) and younger leaves with smaller diameter vessels would tend to freeze at a lower temperature partly due to capillary freezing point depression (Liu et al. 2003).

Kaku and Salt (1968) also observed an age-dependant freezing pattern in conifer needles but attributed the difference to the length of the needles. They assumed that longer needles would have more intrinsic nucleators and would therefore, freeze at a higher temperature. In our study, the oldest leaf was one of the smallest and it always froze first. In fact, we did not observe any relationship between leaf size and freezing point. We propose that the pattern of freezing from outermost (oldest) to innermost (youngest) leaves in wheat is determined by all the factors mentioned above: diffusion of latent heat from freezing of outermost leaves, differences in water content, and anatomical differences between old and young leaf tissues that affect ice propagation, particularly in xylem vessels.

### Ice formation initiated in basal portions of the plant

**Fig. 6:**
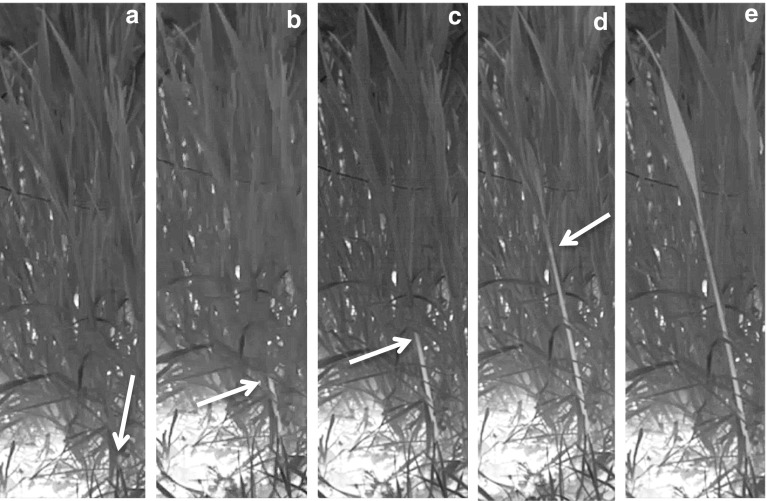
Five frames from a high-definition recording (SV3) of wheat plants under natural conditions showing freezing progressing from the bottom of the plant to the top. This, in spite of the canopy, near the top of the plants, being around 4 °C colder than the soil (Fig. 2b). The arrows show the progression of freezing. The arrow in **a** is the position on the plant where freezing was first visible. Arrows in **b** through **d** show the progression of freezing; **e** shows the entire plant frozen. Freezing in all the plants observed was initiated at the base of the stem and progressed to the top

One of the most surprising observations was that freezing in wheat plants was consistently initiated at the bottom of the plant and progressed to the top (Fig. 6, SV2, SV3). This was despite air temperatures that were as much as 5° colder than the base of the plant near the soil. This observation was decidedly counterintuitive since freezing has long been assumed to begin somewhere in the upper part of leaves (Single 1964; Olien and Smith 1981) and then spread to the lower regions of the plant. Pearce and Fuller (2001) indicated that ice was initiated in hydathodes of barley leaves; however, this was in isolated plants under controlled freezing conditions. Pearce and Ashworth (1992) suggested that freezing was initiated by ice entering the leaf through stomata. Fuller et al. (2007) reported the ability of wheat plants in the field to supercool and avoid freezing but no mention was made of the sites of ice formation or the progression of ice propagation. Basal initiation of freezing in wheat grown and frozen under controlled conditions was previously reported by Livingston et al. (2016) and the results of the current study confirm this observation under natural frost episodes in the field (Fig. 6, SV2, SV3). All plants in the current study froze from the bottom up (SV2, SV3), even when thermocouple readings, as well as IR observations, indicated that the soil was not frozen (Fig. 2b, SV2, SV3). In many cases the soil did not appear to be frozen but, it is possible that a thin layer of water on some surfaces had frozen and could have served as an ice nucleator, initiating ice formation at the base of stems.

Freezing of leaves in grain crops from the top down was only observed once or twice (less than 1% of all observations over hundreds of tests) in studies conducted from 2013 to 2017. Even when leaves were cut, exposing interior tissues to the air, freezing began from the bottom of the plant (not shown). When plants were sprayed with plain water (containing no ice-nucleating substance), freezing still occurred from the bottom up. In fact, water droplets on the surface of leaves often froze after leaves themselves froze (Livingston et al. 2016); this was in contrast to observations made by Fuller et al. (2007) where wheat plants sprayed with INA bacteria froze at the warmest temperature followed by plants sprayed with water. Dry plants were the last to freeze. No information was provided, however, about the initial site of ice nucleation or the progression of ice propagation. Pearce and Fuller (2001) also indicated the ability of INA bacteria to induce ice formation in barley leaves and Wisniewski et al. (2002) have noted the ability of dry, herbaceous plants to supercool to lower temperatures before freezing than plants sprayed with either water or water containing INA bacteria.

Hacker and Neuner (2007) found that nucleation in stems of *C. camphora* always occurred first in stems and then progressed into leaves. They suggested that the presence of large quantities of water with low solute content in vessels within stems would promote nucleation and the spread of ice. Stier et al. (2003) demonstrated that freezing in two different turf grasses began in roots and proceeded through the crown into the leaves (Stier et al. 2003). If low solute content favors nucleation within a xylem conducting element, then one could hypothesize that the structural characteristics of the xylem tissue would influence the ability of ice to propagate up through a leaf or stem (Kuprian et al. 2016). Larger diameter vessel elements, characteristic of older leaves with more mature xylem tissues (e.g., metaxylem vs. protoxylem) would favor rapid ice propagation into a leaf and hinder propagation in younger leaves. Moreover, Pearce and Fuller (2001) saw no evidence of lateral ice propagation from one leaf to another even when leaves were in contact with each other. Hacker and Neuner reported similar results when gramenoid tussocks were frozen (Hacker and Neuner 2008). Lateral propagation of ice was not observed in the present study either.

Preliminary studies were conducted to determine the nucleation temperatures of apoplastic extracts to determine the basis for initiation of ice in basal portions of the stem. Our preliminary data indicated the presence of compounds in the apoplastic extracts that were particularly effective at inducing ice to form at warm, sub-zero temperatures (− 3 °C) and that crown tissues had higher ice nucleation activity than leaves (data not shown). Pummer et al. (2015) and Ashworth and Kieft (1995) described numerous water-soluble, ice nucleating macromolecules found in fungi, bacteria, and pollen that are effective nucleators at warm, sub-zero temperatures. In addition, Brush et al. (1994) identified several proteins, carbohydrates, and phospholipids that were effective ice nucleators in leaves of rye. Wisniewski et al. (2014) provided a comprehensive list of intrinsic ice nucleators that have been identified in plants. Further research is being conducted on the apoplastic extracts from wheat to characterize these putative nucleators.

**Fig. 7:**
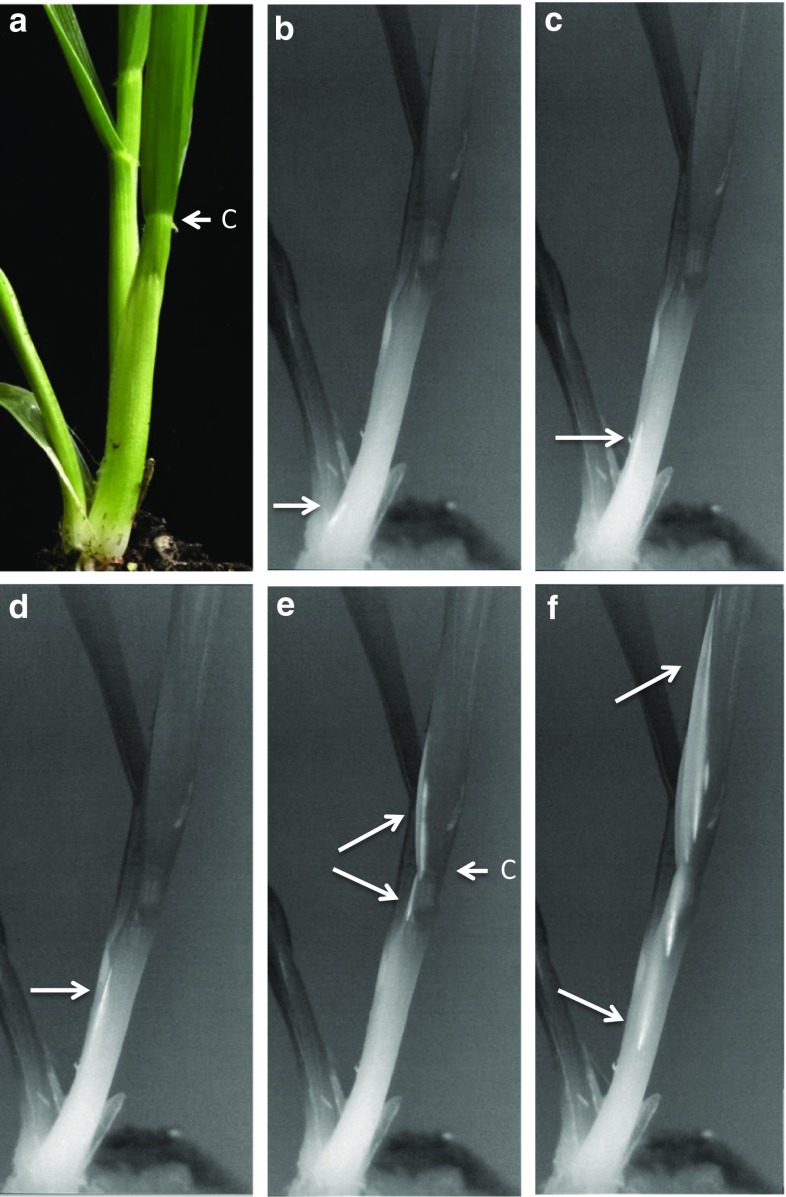
Multiple frames from a close-up, high-definition recording of the base of a wheat plant showing the initial, stage 1 freeze event beginning at the base of the stem just above the roots. **a** The plant in an unfrozen condition with the oldest leaf of the secondary tiller facing the viewer. The arrow at “c” identifies the collar of the oldest leaf where the sheath and leaf meet. **b**–**d** Freezing is shown (arrow) progressing in what appears to be a single vascular bundle from the bottom of the plant upwards until meeting the collar. **e**, **f** Once reaching the collar, freezing spread to other vascular bundles and progressed both up and down the leaf in vessels that had apparently not yet frozen. The reader is encouraged to view the video to better visualize this freeze event and to observe the two other tillers freezing in an identical manner

Closer observations of the base of the stem under controlled conditions (Fig. 7 and SV5) indicated that freezing in leaves originated just above the crown at the base of the stem and within a single vessel bundle that was probably the midrib (Fig. 7b–d). When the ice front reached the collar of the oldest leaf, it spread upwards as well as down through vessels that had apparently not yet frozen (Fig. 7e, SV5). An identical pattern was observed in the other two tillers; this freezing pattern is much better visualized in the supplemental video (SV5). This implies that there is a restriction or change in the continuity of the vessels at the junction of the sheath and leaf base that inhibits further immediate ice propagation. Stier et al. (2003) reported a similar observation in perennial ryegrass and suggested that the delay in the progression of ice propagation through the collar was the result of “some sort of barrier”. Single (1964) reported that the stem and rachis nodes of field-acclimated wheat inhibited the spread of ice crystals. It seems reasonable to assume that vessels within the collar undergo a transition from the sheath into the leaves, perhaps similar to the transition that vessels undergo within nodes of grasses (Hitch and Sharman 1971).

#### Freeze injury to leaf tips

It is a common assumption that leaf tips are injured after a freezing event because they are exposed to lower air temperatures and therefore, freeze while other portions of the leaf do not. Our observations using high-definition IR imaging, however, indicate that although leaf tips were generally about 2 °C cooler than the rest of the plant, ice formation was never restricted to just leaf tips. The entire leaf froze in all plants that exhibited subsequent leaf-tip burn after thawing (not shown), under both controlled and natural conditions. This indicates that the tips of leaves have a lower tolerance to whatever freezing conditions were encountered by the entire leaf. It is possible, since the leaf tips were generally 2–3 °C cooler (more supercooled) than the lower portion of the leaves, that when they did freeze, the extent of supercooling created a more destructive non-equilibrium freeze event in the cells of the leaf tips. This could induce intracellular ice formation in some cells; however, a more detailed analysis of freezing in leaf tips would be necessary to confirm this.

How specific tissues might withstand a non-equilibrium freeze following supercooling is an important consideration since cells in different parts of the plant supercooled to a different extent. It is reasonable to assume that the degree of supercooling can play a role in determining whether a cell survives a non-equilibrium freezing event. Apparently, even though some leaves supercooled to a greater extent than others, there was a difference in the capacity to withstand a non-equilibrium freezing event when it did occur. For example, the oldest leaves supercooled to the least extent (i.e., they were the first leaves to freeze), and yet they were killed by a relatively mild freeze (− 4 to − 6 °C). A supercooled system is metastable and the greater the extent of supercooling the more water that must move from the cytoplasm to the site of extracellular ice when freezing occurs so that the system can come into equilibrium (Burke et al. 1976). The inability of water to be removed rapidly from cells would increase the chances of a lethal, intracellular freezing event to occur. In this regard, Gusta et al. (2004) observed that acclimated canola leaves could tolerate a higher level of supercooling accompanied by subsequent ice formation than non-acclimated leaves. This suggests there may be mechanisms to protect some leaves from non-equilibrium freezing that are missing in others. Griffith et al. (2005) also reported that older leaves of rye plants are the least hardy and that only young or newly formed leaves have the capacity to cold acclimate. The extent of supercooling was greater in the youngest leaves with some freeze events occurring at − 18 to − 20 °C, without any subsequent injury. This suggests there are mechanisms that allow plants to withstand severe non-equilibrium freezes which should be considered when evaluating the cold hardiness of different plant species and/or genotypes within a species.

### Stages of freezing

**Fig. 8:**
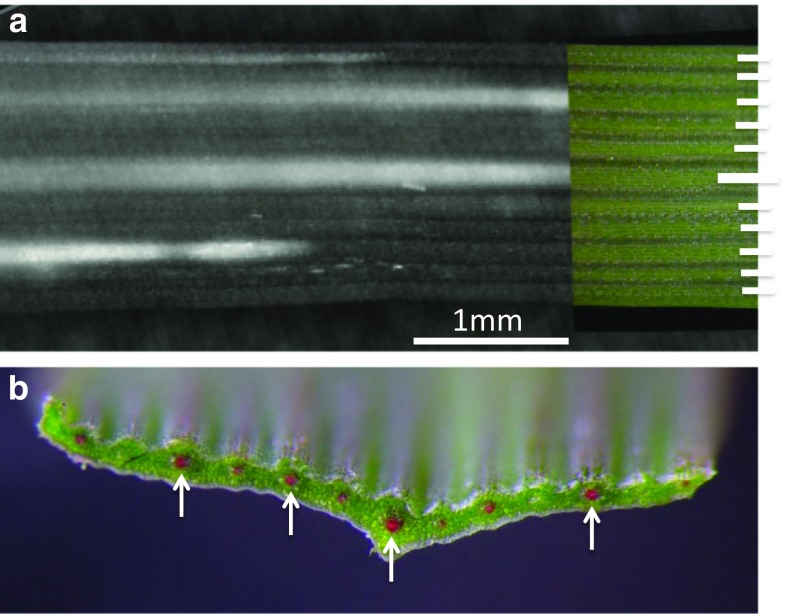
A single frame from a close-up, high-definition recording (SV5) midway along the length of the oldest leaf from the primary tiller (leaf 3 from Fig. 1b) of an intact plant. Note that freezing is within the vascular bundles at this point in the freeze event. The inset to the right in **a** is part of a visible image of the leaf before it was frozen and is aligned with the infrared frame. The short, white horizontal bars on the right side of the inset indicate where the vascular bundles are located. The longest bar is the midrib of the leaf. **b** A fresh wheat leaf, cut in cross section with vascular bundles infused with Safranin to show their precise location; arrows point to the four largest bundles. The reader is encouraged to view supplemental video 5 to better visualize this demonstration of freezing in the vascular bundles of a wheat leaf

**Fig. 9:**
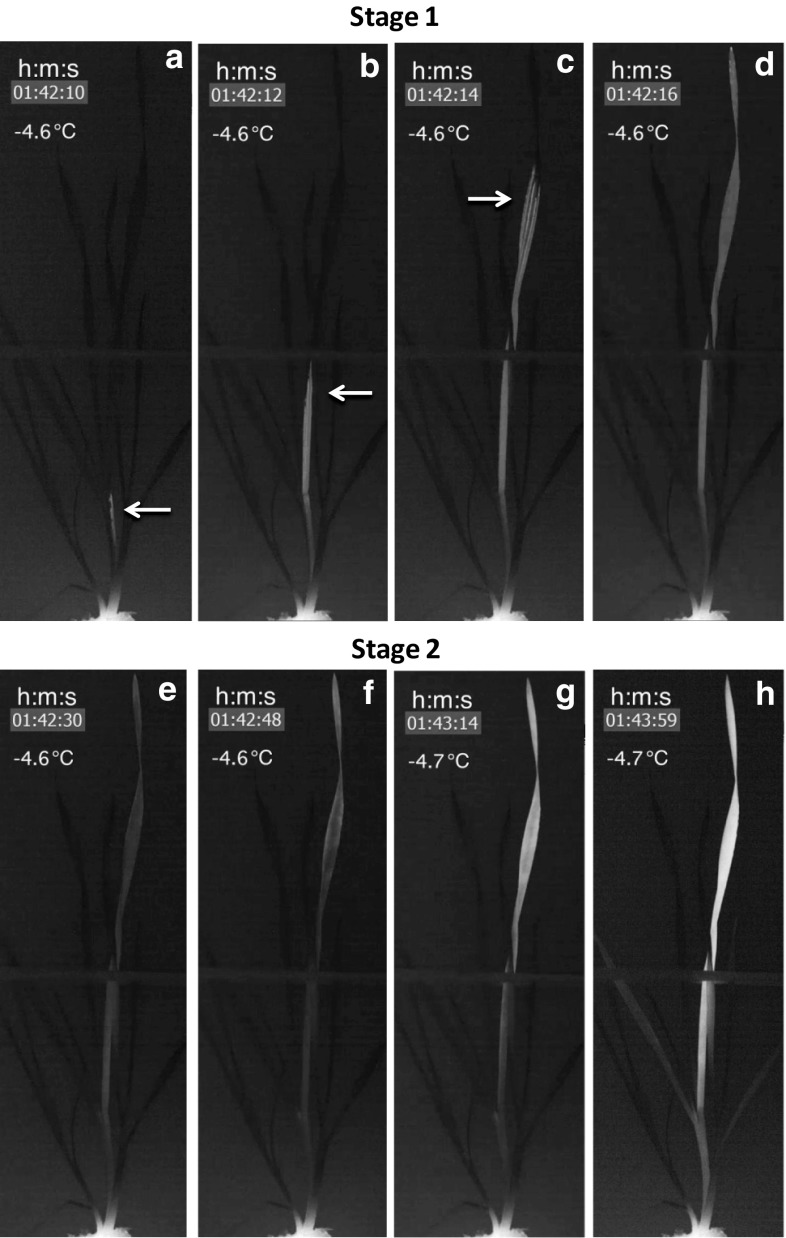
Oldest leaf of the primary tiller (leaf 3, see Fig. 1b) of a cold-acclimated wheat plant shown freezing under controlled conditions in two stages. **a**–**d** Stage 1 freezing was always much faster than stage 2 or 3. In this case ice progressed from the bottom of the plant to the top of the leaf in 6 s. Note the differentiation of vascular bundles in C (arrow). **e** Shows the leaf almost coming to equilibrium with the background temperature after stage one freezing and just prior to stage 2 freezing. **f** Shows the beginning of stage 2 freezing which was brightest at the top of the leaf but overall appeared to freeze in a more uniform manner throughout the leaf. This freeze event spread over the entire leaf within a minute (shown in **g** and **h**) but took about 10 min to come to equilibrium with the surrounding temperature; the complete freeze event can be observed in supplemental video 6

Freeze events that were non-lethal were observed in cold-acclimated wheat plants exposed to freezing temperatures down to about − 5 or − 6 °C under controlled conditions. These freeze events always occurred in two stages, with an initial rapid freeze occurring in vascular bundles (Figs. 8, 9) and probably apoplastic spaces. After the first freeze event reached the tip of the leaf, a brighter (presumably involving more water) exotherm spread throughout the leaf (Fig. 9h, SV6, SV7). Latent heat from the first stage freezing event dissipated within seconds while it took nearly 5 min (depending on the temperature) for heat from the second stage to dissipate. The transition from stage 1 to stage 2 is much easier to observe in the supplemental videos (SV6, SV7). The longer time to reach equilibrium after the second stage of freezing indicates that more water froze than in the first stage.

Pearce and Fuller (2001) also described two stages of freezing in barley. They suggested that the first stage represented the freezing of apoplastic water, and that the second stage represented freezing of water that moved from the cell to sites of extracellular ice. The removal of cellular water was necessary for the cell to come to equilibrium with the temperature of extracellular ice. The lower the temperature, the more water that must be removed for the system to remain in equilibrium and so the cells do not freeze, but instead dehydrate. This slower freezing process that occurs at small displacements from equilibrium is referred to as equilibrium freezing (Olien 1974) and hence freezing stress is often characterized primarily as dehydration stress (Wisniewski et al. 2014).

Pearce and Fuller (2001) stated that freezing appears to occur in “strips” in barley leaves but did not indicate that xylem vessels were the main conduit. This was most likely due to the low-resolution image provided by the IR camera that made it difficult to discern the initial pathway of ice propagation. Our observations with much higher definition IR images (Fig. 8 and SV6) indicate that freezing in wheat begins in xylem conduits and then spreads throughout the mesophyll. Hacker and Neuner (2007) suggested that the two stages of freezing do not represent separate freezing events but “reflect freezing in different tissues”. They indicated that stage 1 freezing in leaves of the subtropical tree, *Cinnamomum camphora,* occurred in xylem conduits, lasting approximately 1.5 min and that stage 2 represented mesophyll freezing which lasted 17 min (Hacker and Neuner 2007).

Antikainen et al. (1996) reported that glucanase and chitinase like proteins (both with antifreeze activity) were associated with bundle sheath cells surrounding vascular bundles and speculated that the presence of these antifreeze proteins (AFPs) slowed secondary ice nucleation of leaves from xylem vessels. In our study, plants were not analyzed for AFP’s but results reported by Antikainen et al. (1996) provide a reasonable explanation for the delay between freezing in xylem and freezing in the rest of the leaf that we observed.

#### Third stage of freezing

While the exact initiation site of stage 1 and stage 2 freezing may differ in different species, all reports on freezing observed with infrared thermography agree that there is an initial rapid freezing event that occurs in stage 1. This is followed by a more substantial freeze event (stage 2) and is most likely a result of water freezing as it moves out of cells to sites of extracellular ice (Hacker and Neuner 2007). In this study when wheat plants underwent both stage 1 and 2 freezing and were subsequently thawed, the leaves completely recovered (not shown). However, after undergoing stage 1 and 2 freezing, when plants were slowly frozen 6° or 8° colder and then thawed, even though no additional freezing events were observed, injury was obvious and leaves eventually died (not shown). Therefore, we suggest that a third stage of freezing exists after stage 1 and 2 and over a range of temperatures. This slow freezing over a range of temperatures would correspond to equilibrium freezing and is probably the result of dehydration stress which would not be detectable with IR thermography.

Stage 3 freezing could be an extension of stage 2 but the temperature range over which it occurs will likely differ depending on the species, and extent of acclimation. Whereas stage 1 lasted for a few seconds to a minute or more, stage 2 lasted for several minutes, and stage 3 transpired over several hours. These durations occurred in the context of the temperature of the system being lowered by 1–2 °C/h. It is reasonable to assume that the rate of freezing, as well as the lowest temperature the plant is exposed to, would influence the duration of each stage.

Hacker et al. (2008) discuss a two stage freezing process and used fluorescence measurements in conjunction with infrared differential thermal analysis in mesophyll cells of several plant species and showed that dehydration stress had significantly different effects on mesophyll cells depending on the species. Olien (1974) discussed various forms of freezing stress and postulated that non-equilibrium freezing occurs down to about − 10 °C. Dehydration and/or adhesions, that predominate at lower temperatures (Olien and Smith 1977) would not be detectable with IR thermography and could be considered stage 3 freezing. In the present study, stage 1 and 2 were non-lethal, while stage 3 was lethal.

Interestingly, the three stages of freezing occurred at very different temperatures within the same plant, depending on which leaf was considered. For example, in older leaves, stage 1 and 2 (a non-equilibrium freeze) occurred at a warmer temperature and if the plant was frozen to a lethal temperature (around − 18 °C), stage 3 (an equilibrium freeze probably inducing dehydration stress) would then have occurred over a longer time period than for a younger leaf in which stage 1 and 2 freezing occurred later. This means that younger leaves would encounter more supercooling and a more severe non-equilibrium freeze. Very little if any equilibrium freezing (resulting in dehydration stress) would occur in younger leaves. In experiments with rye (not shown) stage 1 and 2 freezing were routinely observed to occur in the innermost (youngest) leaves at temperatures between − 16 and − 18 °C; the LT_50_ of the rye cultivar used was around − 20 °C.

In wheat, the oldest leaf was consistently observed to be dead in plants exposed to temperatures at which the rest of plant was uninjured. This suggests that the longer period of time during which older leaves were exposed to dehydration stress was more destructive to leaf tissues than the more severe non-equilibrium freeze in younger leaves. This may be the basis for the difference between levels of injury in an acute freezing stress (short duration) vs. a chronic stress (long duration). Gusta and colleagues have frequently noted that plants subjected to a short, controlled freezing stress (acute stress) have a lower LT_50_ than plants subjected to a long duration freezing stress (days or weeks) at a warmer temperature (Gusta et al. 2001; Waalen et al. 2011).

### Leaf histology at freezing stages 1, 2 and 3

To better understand how each stage of freezing impacted leaves, portions of the oldest leaf on the primary tiller were immersed in fixative at the same temperature as the leaf, immediately after each stage of freezing. The purpose of this technique is to fix changes in anatomical configurations caused by ice formation within tissues. Empty spaces or voids in freeze-fixed tissues that are not present in unfrozen tissues are theoretically regions where ice had formed (Livingston and Tuong 2014). This freeze-fixation technique was originally developed for use in examining ice within rodent hearts (Mackenzie et al. 1975) and was adapted for use in observing ice formation in forsythia flower buds (Ashworth 1990) and in frozen oat crowns (Livingston and Tuong 2014).

**Fig. 10:**
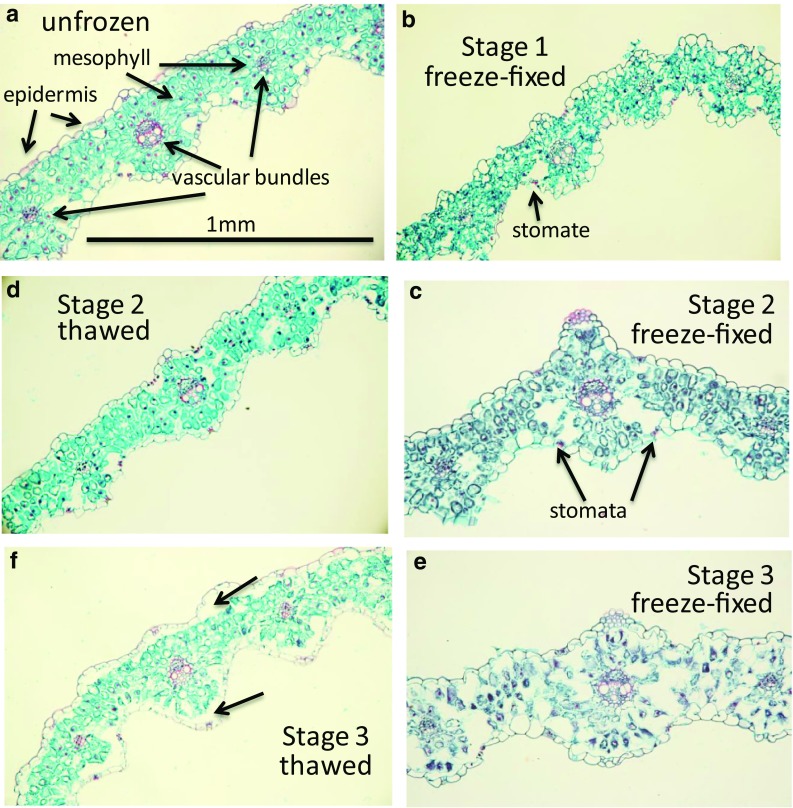
Twenty micron thick, triple-stained (Safranin, Fast Green, Orange G) cross sections of the oldest leaf from the primary tiller (leaf 3, see Fig. 1b), midway between the tip of the leaf and the leaf collar. **a** An unfrozen section showing epidermis, mezophyll and vascular bundles. **b** Cross section of a leaf that was freeze-fixed immediately after stage 1, but before stage 2 began, when the leaf had reached equilibrium with the surrounding temperature. When thawed, this leaf showed no visible difference under our histological conditions from the freeze-fixed section (not shown) **c**. Cross section from a different plant but from the same position of leaf 3; this leaf was freeze-fixed after stage 2 freezing had equilibrated with the surrounding temperature. Note the minor difference between stage 1 (**b**) and stage 2 (**c**) in overall appearance. Also note the increased size (compared to the unfrozen leaf in **a**) of the sub-stomatal cavities suggesting the accumulation of ice. **d** Upon thawing after stage 2 freezing, the leaf appeared almost identical to the unfrozen control leaf. This is not surprising since stage 2 freezing occurred at around − 5 °C for this leaf and the leaf always appeared undamaged up to 3 weeks after thawing. **e** Cross section of leaf 3 that was freeze-fixed after the leaf had been frozen to − 20 °C. The wheat cultivar we used has never survived a test when frozen to this temperature. Note the shrunken appearance of mesophyll cells as compared to unfrozen and stage 1 and 2 frozen plants. Stage 3 freezing occurred while the plant was frozen from − 5 to − 20 °C over an 8-h period. No visible freeze events were observed in any IR recordings during the duration of this stage of freezing. **f** Upon thawing, under our histological conditions, the mesophyll cells all appeared to regain turgor. It is not known, however, whether the cells are functional. Interestingly the epidermis appeared to be separated from the mesophyll on both the top and bottom of the leaf. The arrows show the space between the epidermis and mesophyll (compare to **a**, as well as **d**)

There was very little difference between stage 1 and stage 2 frozen leaves (Fig. 10b, c) using freeze-fixation. As compared to unfrozen controls (Fig. 10a), stage 1 and 2 frozen leaves appeared to have somewhat larger voids within the leaf, particularly beneath stomata. This is not surprising since the sub-stomatal cavity is an air filled space that can provide a region for ice to accumulate unhindered as the tissue freezes. Pearce and Ashworth (1992) stated that ice in wheat leaves was located sub-epidermally and was found to accumulate in the sub-stomatal cavity. While very little disruption of cells in the vascular bundles was apparent, the mesophyll cells were somewhat distorted as compared to unfrozen tissues (Fig. 10, compare b, c to a). The lack of significant difference between frozen and unfrozen controls supports the observation that this leaf, at least, will fully recover if thawed after stage 2 freezing. This means that despite a significant non-equilibrium freezing event at around − 4 °C (Fig. 4c) cell walls and membranes were not permanently damaged.

Stage 3 freezing (− 20 °C) always killed the oldest leaf on the primary tiller (leaf 3) and as a consequence exhibited the biggest histological difference from controls and from stage 1 and 2. Freeze-killed leaves were characterized by considerable shrinking of mesophyll cells throughout the portion of the leaf sampled (Fig. 10e compared to 10a). Mesophyll cells in these sections resembled the descriptions given by Pearce and Ashworth (1992) in a freeze-fracture study of wheat leaves frozen to − 9 °C. They describe the presence of extensive sub-epidermal ice throughout the leaf, especially in sub-stomatal cavities, with considerable shrinking of the mesophyll, but without the occurrence of injury (Pearce and Ashworth 1992). In our study, upon thawing, most of the mesophyll cells retained their turgor and appeared similar to mesophyll cells from stage 2-thawed leaves (Fig. 10f). Interestingly, in stage 3 the epidermis separated from the mesophyll leaving an empty space (Fig. 10f, arrows). While this was commonly observed in all the leaves sampled, the space between the epidermis and mesophyll was more extensive in some leaves than others. Epidermal separation was not apparent in the stage 3 freeze-fixed sections (Fig. 10e) so it is not likely a result of ice lens formation during freezing as described by Hacker and Neuner (2007) in *Buxus sempervirens*. While Pearce and Ashworth (1992) found ice between the epidermis and mesophyll they do not mention a significant separation between the two tissues.

Currently, not enough information is available to speculate what caused the epidermis to separate from the mesophyll nor if the mesophyll or epidermis was still functional after thawing. When the plant was returned to optimal growing conditions this leaf shriveled completely within 24 h and never recovered so it is not likely that the epidermis, at least, survived the freeze. Even though the mesophyll cells beneath the epidermis seemed to regain turgor and did not appear ruptured we do not know if they were alive after stage 3 freezing.

### Firefly freezing

**Fig. 11:**
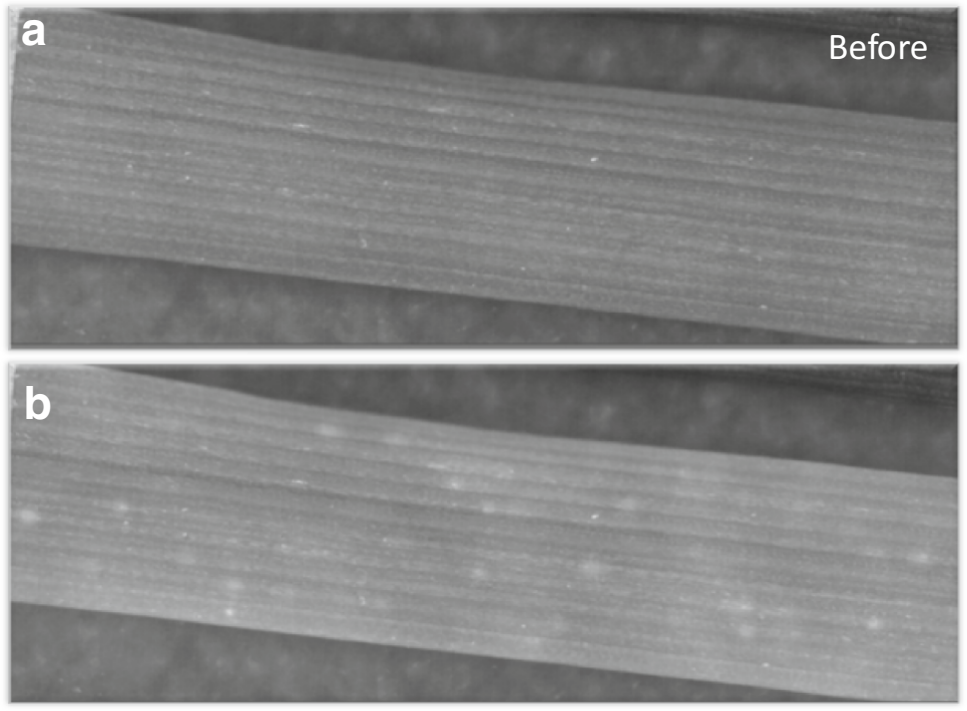
**a** A portion of the oldest leaf from the primary tiller (leaf 3) prior to stage 1 freezing. **b** A single frame from a high-definition recording of a wheat leaf after stage 1 freezing and leading into stage 2 showing freezing of individual cells that resembled firefly activity. This freeze event was only detectable with the high-definition camera used in this study. It is likely that the individual cells freezing are those in the epidermis. A single frame cannot capture the true nature of this freeze event; therefore, the reader is strongly encouraged to view this event in SV5

One curious freeze event not observed until we used the high-definition FLIR SC8303 camera was after stage 1 but prior to stage 2. In this event we observed what appeared to be hundreds of individual cells rapidly freezing in a random pattern resembling firefly activity (Fig. 11, SV6). This “firefly freeze” lasted for about 5 or 10 min and eventually progressed into stage 2 freezing. It is possible that water from individual cells was quickly released and then frozen. However, leaves that were frozen to stage 2 and then thawed showed no sign of cellular rupture (Fig. 10d). This is not surprising since under our conditions, stage 1 and 2 freezing was never lethal. Palta et al. (1977) described recovery in onion cells that had been frozen at a non-lethal temperature (− 4 °C) and indicated that while they initially showed signs of injury (using electrolyte leakage as an assay), they eventually recovered completely. This indicated that if in fact, membrane injury caused by freezing did occur, cells were able to recover and maintain functionality. Our evaluation of injury was made by observing leaves 3 weeks after thawing. Under our conditions the oldest primary leaf (leaf 3) in which firefly freezing was observed always recovered completely as long as it was thawed prior to stage 3 freezing.

It is generally accepted that intracellular freezing will kill a plant cell (Pearce 2001), However, we have difficulty explaining what we observed (Fig. 11 and SV6) other than by intracellular freezing since we did not observe cellular rupture either in freeze-fixed (Fig. 10c) or leaves that were thawed after stage 2 (Fig. 10d). We suspect most of the freezing recorded in SV6 occurred in epidermal cells since the focal plane of the lens used was very narrow (20 μm) and freeze-signatures from mesophyll cells beneath the epidermis would probably have been larger and more diffuse. Research is ongoing to determine an explanation for this freeze event.

## Conclusion

Infrared thermography has clearly provided an unprecedented means to study freezing in plants. Since its first use, many potential investigative questions have been considered, regarding points of inoculation, speed of propagation, as well as identifying specific tissues that are able to supercool. In this study, using a significantly higher definition camera than has been used before, we identified several misunderstandings regarding freezing in small grains that have been accepted as fact for some time. Most notably, we determined that freezing in grasses does not begin at the tips of leaves, even though they are significantly colder than the base of plants or the soil. It was informative to discover that the entire plant does not freeze at once and that at any given temperature much of the plant can remain supercooled. Or that the plant can undergo devastating freeze events without the soil freezing at all. While age-dependent freezing has been described in other species and in grasses it was still enlightening to discover what is a logical sequence of freezing in wheat from oldest to youngest leaves. Is the plant sacrificing the oldest leaves to give the younger “generation” a better chance at survival? The higher definition camera was able to definitively show for the first time that freezing in wheat leaves is initiated in vascular bundles of the leaf, most likely in xylem vessels. And following this initial freeze, individual cells are frozen in a stochastic manner which leads to a second stage of freezing that has a distinctly different freezing pattern than either the first stage or the putative dehydration stage that follows. It was also encouraging to discover that many of the freezing methods long used under laboratory conditions do in fact accurately reflect what happens in the field. These and other findings reported here underscore the importance of observational research so that effective metabolic and genetic studies can be performed and practical breeding strategies can be developed.

### *Author contribution statement*

DPL: conceived of the study, wrote the manuscript. TDT: performed histological analyses. JPM: provided germplasm and consultation on experimental design. LVG performed experiments on apoplastic extracts and provided interpretation of results. IW performed statistical analyses and helped write the mss MEW: Provided camera and expertise on its use and helped write the manuscript.

## Electronic supplementary material

Below is the link to the electronic supplementary material.

SV1 Wide angle view of wheat plants in a vegetative state under natural conditions, recorded with a medium-resolution infrared camera during the night of January 18, 2016. Time is lapse-time from the start of the recording in hours:minutes:seconds. Soil and air temperatures were taken from thermocouple wires placed 1cm below the surface of the soil and in the middle of the plant canopy near the tops of the plants. Note individual plants freezing that are seen as faint flashes of light throughout the plot. The video was sped up considerably (see time signature) to allow viewing of multiple freeze events in a reasonable length of time (MOV 11562 kb)

SV2 The same freeze event as SV1, under natural conditions, but with a second medium-resolution camera positioned closer to individual plants to see individual leaves freezing. As in SV1, time is lapsed from the beginning of the recording in hours:minutes:seconds (MOV 10986 kb)

SV3 High-definition infrared recording of wheat plants in a reproductive phase of growth under natural conditions during the night of March 15, 2017. Waves of warmer air moving through the canopy are visible when the video is time-compressed as shown here. Note plants freezing independently with some plants remaining supercooled down to –8°C and finally freezing early in the morning. Also note that freezing always began at the bottom of the plant and progressed to the top (MOV 46674 kb)

SV4 Wheat plant frozen under controlled conditions and recorded with a high-definition infrared camera. This video shows typical age-dependant freezing from the oldest leaves freezing first (at -2°C) to the youngest (leaf 6) at -5°C. Note that under our growing conditions at least 2 more leaves should be present inside leaf 6; these leaves had not frozen by the end of the test (MOV 23719 kb)

SV5 Close-up recording of the base of a wheat plant freezing under controlled conditions with a high-definition infrared camera. Note freezing beginning at the base of the secondary tiller. A black sliding bar shows where freezing was first visible at the base of the leaf and follows the progression of freezing partway up the tiller. Note that freezing moves upwards in what appears to be a single vessel bundle until reaching the collar. Also note that the other 2 tillers freeze later in an identical manner (MOV 21355 kb)

SV6 High-definition recording of freezing under controlled conditions of a portion of the oldest leaf on the primary tiller (leaf 3) of an intact plant. Note, this event is in real-time and the initial freeze (stage 1) progresses very quickly. Also note that about 1 min after the beginning of the recording, individual cells (likely within the epidermis) began to freeze in what appeared to be a random manner covering this portion of the leaf. This firefly- like freezing led into the second stage of freezing in which the entire leaf turned a significantly lighter color indicating widespread freezing (MOV 16341 kb)

SV7 Wheat plant under controlled conditions recorded with a high-definition infrared camera showing stage 1 and stage 2 freezing. Leaves were not killed following this 2-stage freezing event, but would have been killed after stage 3 freezing (MOV 16240 kb)

## Abbreviations

IRT: Infrared thermography
INA: Ice-nucleating activity
MRI: Magnetic resonance imaging
